# The role of KLRG1: a novel biomarker and new therapeutic target

**DOI:** 10.1186/s12964-024-01714-7

**Published:** 2024-06-19

**Authors:** Yakun Zhang, Shuang Chen, Xinyi Tang, Yu Peng, Tingting Jiang, Xiaomei Zhang, Jun Li, Yao Liu, Zailin Yang

**Affiliations:** 1 School of Medicine, Chongqing University, Chongqing, 400030 China; 2 Department of Hematology-Oncology, Chongqing Key Laboratory of Translational Research for Cancer Metastasis and Individualized Treatment, Chongqing University Cancer Hospital, Chongqing, 400030 China

**Keywords:** KLRG1, Immune checkpoint proteins, Biomarker, Therapeutic target, Tumor immunotherapy

## Abstract

Killer cell lectin-like receptor G1 (KLRG1) is an immune checkpoint receptor expressed predominantly in NK and T-cell subsets that downregulates the activation and proliferation of immune cells and participates in cell-mediated immune responses. Accumulating evidence has demonstrated the importance of KLRG1 as a noteworthy disease marker and therapeutic target that can influence disease onset, progression, and prognosis. Blocking KLRG1 has been shown to effectively mitigate the effects of downregulation in various mouse tumor models, including solid tumors and hematologic malignancies. However, KLRG1 inhibitors have not yet been approved for human use, and the understanding of KLRG1 expression and its mechanism of action in various diseases remains incomplete. In this review, we explore alterations in the distribution, structure, and signaling pathways of KLRG1 in immune cells and summarize its expression patterns and roles in the development and progression of autoimmune diseases, infectious diseases, and cancers. Additionally, we discuss the potential applications of KLRG1 as a tool for tumor immunotherapy.

## Background

KLRG1 is an inhibitory lectin-like type II transmembrane glycoprotein receptor characterized by an extracellular c-type lectin structural domain, a transmembrane structural domain, and an inhibitory motif for the cytoplasmic immunoreceptor tyrosine-based inhibitory motif (ITIM) [1]. To date, this receptor has been described in diverse subsets of lymphocytes, including natural killer (NK) cells, as well as distinct subsets of T-cells, such as CD8^+^ T-cells, CD4^+^ T-cells, and regulatory T-cells (Tregs) [2–5]. The extracellular domain of KLRG1 on immune cells can be assessed via immunofluorescence staining and flow cytometry using KLRG1-specific antibodies, enabling the quantification of its expression level [6, 7]. As a ligand of KLRG1, cadherin is widely expressed on antigen-presenting cells (APCs) and tumor cells and binds to the extracellular domain to achieve signal transduction [8, 9]. Signaling through the binding of KLRG1 to cadherin occurs only secondarily to the successful activation of lymphocytes via the T-cell receptor (TCR) with its cognate major histocompatibility complex or other activating receptor ligand, followed by the phosphorylation of downstream proteins, including AKT or AMP-responsive protein kinase (AMPK) [10–12]. The primary function of KLRG1 is to provide stimulatory (costimulatory) and inhibitory (coinhibitory) signals, thereby regulating the activation and proliferation of immune cells to self-antigens and foreign antigens and participating in cell-mediated immune responses [13]. The most characteristic inhibitory functions attributed to KLRG1 include the induction of immune cell death or exhaustion through autophagy, the suppression of cytotoxicity, and the inhibition of cytokine production [9, 14].

Recent advances have shown the crucial role of KLRG1 in the pathogenesis and progression of autoimmune disorders, infectious diseases, and malignancies, underscoring its potential utility as a promising immune cell marker for disease prediction, diagnosis, and prognostication [15, 16]. Nevertheless, the expression levels of KLRG1 and the signal transduction pathways in which it is involved exhibit variation among distinct immune cell types [5, 10–12, 17], suggesting diverse regulatory mechanisms and clinical implications in different disease states. In the context of human physiology, KLRG1 has been subjected to thorough investigation across a spectrum of disease states, spanning expedited immune responses to malignant tumor progression. These states include infections, autoimmune disorders, solid tumors, and hematological malignancies (HMs) [16, 18–21]. Ideally, the inhibitory effect of anti-KLRG1 antibody for KLRG1 on immune cells can effectively enhance adaptive immune function or improve vaccine efficacy [11, 22]. Thus, the development of KLRG1-targeted inhibitors, which have emerged as a prominent area in the field of immunotherapy, has accelerated [8, 23–25]. Anti-KLRG1 monoclonal antibodies (mAbs) significantly increase the antitumor activity of immune cells and reduce the worsening of disease in cancer mouse models [8, 24, 26]. Importantly, an anti-KLRG1 mAb (ABC008) for treating autoimmune diseases and hematologic malignancies is already in development [27] and is a novel, promising strategy for disease treatment [8, 24]. Upon the approval of anti-KLRG1 mAbs for therapeutic use, the assessment of KLRG1 levels is poised to assume a critical role as a biomarker in clinical evaluation [16, 28]. Nevertheless, the existing evidence falls short of conclusively addressing specific concerns. First, while KLRG1 expression has been detected in various cell types, including tumor cells, a systematic and comprehensive summary of the potential mechanisms underlying its role is lacking. Second, the differences in the expression and role of KLRG1 in various diseases and the feasibility and clinical significance of KLRG1 as a disease marker have not been summarized. Finally, recent clinical studies of KLRG1 inhibitors have focused only on inclusion body myositis (IBM) and T-cell large granular lymphocytic leukemia diseases, and the feasibility of using KLRG1 as a potential therapeutic target for other diseases still needs to be studied.

Hence, we present a comprehensive review elucidating the distribution, structural attributes, and functional signaling pathways of KLRG1 across various cell types, delineating its multifaceted involvement in assessment of the progression of disease pathogenesis. These findings demonstrate its significance as a biomarker in autoimmune and infectious diseases, as well as its contribution to immune modulation within both solid and hematological tumors. Additionally, we offer an overview of the recent advancements in KLRG1 inhibitor development for tumor immunotherapy, underscored by the promising synergistic efficacy of KLRG1 inhibitors combined with other targeted inhibitors.

## Regulation of immune signaling by KLRG1

### Differences in KLRG1 between mice and humans

KLRG1, known as a mast cell function-associated antigen, was initially characterized in RBL-2H3 mast cells from rats in 1991 [29]. In contrast to that in rats, KLRG1 is not expressed on mast cells in mice or humans [2, 13, 30]. Recent advances indicate that KLRG1 is expressed on immune cells, mainly NK cells, CD8^+^ T lymphocytes, CD4^+^ T lymphocytes, and other T cell subsets of γδ T-cells, follicular helper T-cells, follicular regulatory T-cells and regulatory T-cells in mice and humans [2–5, 17, 31, 32]. In addition, KLRG1 is expressed mainly on mature cells and is expressed at relatively low levels or not expressed on naïve cells, while KLRG1 is heterogeneously expressed on memory T-cells and NK cells [33].

The location and length of the *KLRG1* gene and the structure of the KLRG1 protein differ between mice and humans (Table 1). First, the *KLRG1* gene is located on chromosome 6 in mice and chromosome 12 in humans and is transcribed into mRNA by a promoter, followed by selective splicing of *KLRG1* mRNAs into different forms, and only stable *KLRG1* mRNAs are translated into KLRG1 proteins [34–36]. The extracellular c-type lectin structural domain of the KLRG1 protein is expressed on the cell membrane and undergoes modification processes such as glycosylation and phosphorylation to exert its effects [37]. The mouse *KLRG1* gene (m*KLRG1*) is approximately 13 kb in total length and is composed of five exons and four introns [34]. The length of the rat *KLRG1 (*r*KLRG1)* gene is approximately 13 kb, while that of the human *KLRG1 (*h*KLRG1)* gene is approximately 19 kb. The r*KLRG1* and h*KLRG1* homologs share 89% and 71% similarity with the mouse gene, respectively, with each featuring five exons and four introns [3, 30, 34]. Regarding the KLRG1 protein, there are specific variations in structure between mice and humans, with 57% identity at the amino acid level [38]. Biochemical analyses have indicated that, compared with programmed cell death protein 1 (PD-1), which is expressed mainly as a monomer on the surfaces of cell membranes, mKLRG1 can form monomers, dimers, trimers, and tetramers that are connected by disulfide bonds. In contrast, hKLRG1 exists only as a disulfide-linked dimer [6]. Unlike for PD-1, no study has yet described soluble KLRG1, which may be due to KLRG1 existing mainly as a homodimer. In addition, Hofmann et al. compared the inhibitory capacities of different polymerized forms of KLRG1 by altering the mKLRG1 protein and reported that only disulfide-linked dimeric KLRG1 had a significant inhibitory capacity [39], possibly resulting in a lower inhibitory capacity of mKLRG1 than of hKLRG1. Although there are differences between mKLRG1 and hKLRG1, their binding abilities to cadherin are similar [40]. Therefore, KLRG1-related studies based on mouse models may have some reference value for human disease research, but whether KLRG1 can serve as a biomarker for evaluating disease development or as a therapeutic target still needs to be determined by further clinical study.

**Table 1:** Differences in KLRG1 between mice and humans

	Mouse (mKLRG1)	Human (hKLRG1)	References
Gene Location	chromosome 6	chromosome 12	[34]
Gene Structure	5 exons, 4 introns	5 exons, 4 introns	[3, 30, 34]
Gene Length	13 kb	19 kb	[34]
Protein Structure	monomers, dimers, trimers, tetramers	dimers	[6]
Inhibitory Capability	lower	higher	[39]

### Signaling pathways of KLRG1

**Fig. 1:**
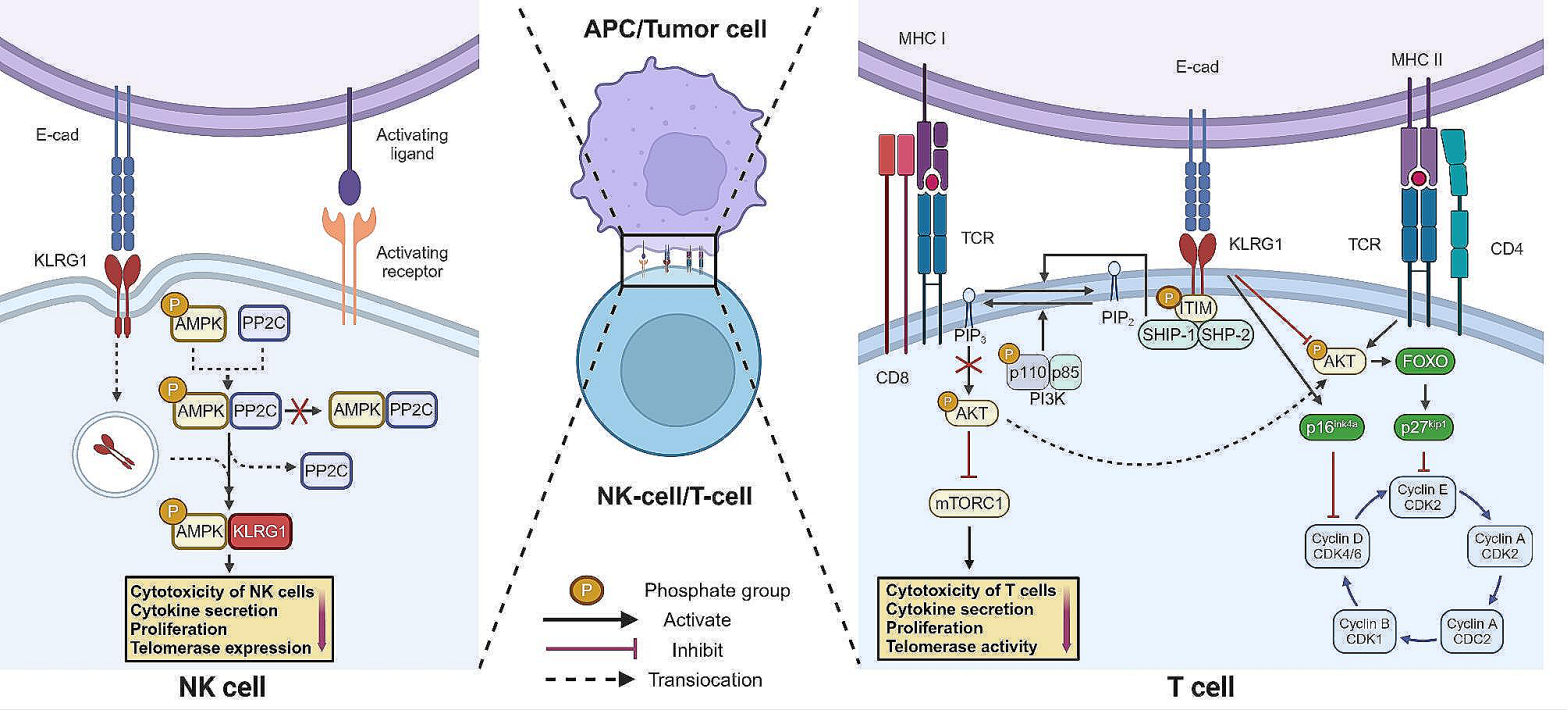
**KLRG1 signaling pathway.** KLRG1 expressed on the surfaces of T-cells interacts with cadherin ligands expressed on the surfaces of cancer cells or APCs, promoting the phosphorylation of the ITIM intracellular structural domain, followed by the recruitment of the tyrosine-protein phosphatases SHIP-1 and SHP-2. In contrast to PI3K, SHIP-1 and SHP-2 inhibit AKT phosphorylation by degrading PIP3 to PIP2, thereby attenuating the activation of the mTOR pathway, leading to reduced T-cell effector function and proliferative dysfunction. In HCV-infected CD4^+^ T-cells, an increase in KLRG1 expression can inhibit AKT phosphorylation, thereby activating the transcription factor FOXO and increasing expression of the cell cycle inhibitor p27^kip1^ or directly activating p16^ink4a^ to inhibit T-cell cycle progression. In NK cells, in addition to affecting the AKT pathway, KLRG1 can also be internalized after binding cadherin ligands, after which it binds directly to AMPK and prevents AMPK dephosphorylation by the protein phosphatase PP2C, which amplifies the activity of AMPK and leads to loss of NK cell function. The figure was created at BioRender.com

The regulatory pathways of KLRG1 in immune cells have undergone extensive investigation, revealing both commonalities and distinctions in the regulatory pathways and functions of KLRG1 across diverse cell types. Functionally, KLRG1 can act as an immune checkpoint receptor to regulate immune cell proliferation and the immune response by binding to its ligand cadherin via the phosphoinositide 3-kinase (PI3K)/AKT pathway or the AMPK pathway [10, 30, 41–43]. Rosshart et al. reported that spatially linked co-engagement of KLRG1 and TCR/CD3 is a prerequisite for KLRG1 function [43]. When cadherin binds to the extracellular domain of KLRG1, ITIM tyrosine is phosphorylated, thereby inhibiting lymphocyte function [44]. mKLRG1 can recognize and bind to three prototypical cadherins, namely, E-cadherin (E-cad), N-cadherin (N-cad), and R-cadherin (R-cad) [9, 45]. The interaction of KLRG1 with E-cad, N-cad, or R-cad increases the activation threshold of NK and T-cells, thereby inhibiting the cytotoxicity of NK cells to prevent damage to tissues expressing these cadherins [9, 46–48]. This mechanism represents a protective measure of the body against excessive immune activity. KLRG1 binds to the N-terminus of the monomeric form of E-cad [46], and this interaction between KLRG1 and E-cad inhibits the proliferation and cytokine production of type 2 innate lymphoid cells (ILC-2s) [49]. The E-cad/KLRG1 pathway plays a significant role in inhibiting the antitumor activities of T-cells and NK cells (Fig. 1) [10, 24, 50]. KLRG1 mainly interacts with cadherin ligands expressed on the surfaces of cancer cells or APCs, subsequently recruiting tyrosine-protein phosphatases (Src homology 2-containing inositol phosphatase-1 (SHIP-1) and Src homology-2-containing protein tyrosine phosphatase 2 (SHP2)) following the phosphorylation of ITIM tyrosine residues within its cytoplasmic structural domain [44, 51]. The effectors SHIP-1 and SHP-2 regulate PI3K function by degrading phosphatidylinositol (3,4,5) trisphosphate (PIP_3_) to phosphatidylinositol (4,5) bisphosphate (PIP_2_) [51]. PI3K, consisting of the regulatory subunit p85 and the catalytic subunit p110, phosphorylates PIP_2_ to produce PIP_3_, which aids in phosphorylating AKT, thus regulating a series of downstream cellular responses, including survival, growth, proliferation, and migration [44, 52–54]. KLRG1 inhibits AKT phosphorylation by inhibiting the PI3K/AKT pathway, thereby attenuating the activation of the mammalian target of rapamycin (mTOR) signaling pathway, resulting in NK and T-cell proliferation dysfunction and reduced effector function [42, 50, 55, 56]. Furthermore, in hepatitis C virus (HCV)-driven CD4^+^ T-cells, KLRG1 can inhibit T-cell cycle progression through the p16^ink4a^/p27^kip1^ pathway [11]. During HCV infection, the increased expression of KLRG1 inhibits TCR-induced PI3K/AKT phosphorylation, which activates the forkhead box O (FOXO) transcription factor and increases expression of the cell cycle inhibitor p27^kip1^, resulting in growth arrest in the G1 phase by repressing the activation of cyclin E and cyclin-dependent kinase-2 (CDK2) [57, 58]. High KLRG1 expression resulting from HCV infection also increases the expression of p16^ink4a^ in CD4^+^ T-cells, which blocks the activation of cyclin D and CDK4/6, leading to growth arrest in the G1 phase [59]. Suppression of the KLRG1 pathway and its downstream signaling molecules in CD4^+^ T-cells restores CD4^+^ T-cell cytotoxicity, providing a novel avenue for enhancing vaccine responses [5].

In highly differentiated human primary NK cells, KLRG1 can also inhibit the function of NK cells through the activation of AMPK, in addition to the classic PI3K/AKT pathway [10, 50]. KLRG1 is internalized after the E-cad/KLRG1 complex is formed and directly binds to AMPK to disrupt AMPK-protein phosphatase 2 C (PP2C) interactions. Subsequently, it inhibits the phosphatase activity of PP2C, preventing the dephosphorylation of AMPK by phosphorylated PP2C. This process enhances AMPK signal transduction rather than inducing de novo kinase activation [60]. Importantly, inhibiting KLRG1/AMPK signaling can prevent AMPK activation and reinstate NK cell cytotoxicity, cytokine secretion, proliferation, and telomerase expression, thereby bolstering immunity in aging individuals and in individuals with malignant tumors [10, 42]. Although human T-cells also express KLRG1 and have the AMPK signaling pathway [61], it is unclear whether KLRG1 plays a similar role in T-cells. In addition, KLRG1 can bind to membrane-bound N-cad, recruiting SHIP-1 and SHP-2, inhibiting NK cell and T-cell function, and inducing cardiac endothelial cell proliferation and angiogenesis [62, 63]. Moreover, KLRG1 can also cause functional depletion of NK cells by binding to soluble N-cad released by circulating tumor cells in a noncontact cell‒cell manner [48, 64]. R-cad binding to KLRG1 plays a similar role [9].

### Effect of KLRG1 on immune cells

Typically, 30% of resting NK cells in mice express KLRG1 [14], while in humans, 60% of healthy adult NK cells express KLRG1 [30]. KLRG1 is a marker of T-cell senescence [65]. KLRG1 is highly expressed in T-cells from senescent patients, and its expression increases with age [42, 65, 66]. In addition, the proportion of highly differentiated T-cells increases in older individuals, and the expression of KLRG1 is correlated with the degree of cellular differentiation [67]. KLRG1 expression increases with the degree of cellular differentiation in NK cells and T-cells and is overexpressed in mature cells, with the highest expression in memory cells and highly differentiated end-stage cells, suggesting that KLRG1 can be used as a marker of lymphocyte differentiation [7]. KLRG1 can also serve as an indicator to distinguish short-lived effector cells from memory precursor effector cells. During acute viral infection in mice, KLRG1 is a marker for short-lived effector CD8^+^ T-cells [68, 69]. KLRG1-positive NK and T-cells have lower proliferative capacities than KLRG1-negative cells [7, 30, 65].

KLRG1 is not only a marker of T-cell senescence [70]. KLRG1 has an inhibitory cytoplasmic ITIM motif and thus may play an inhibitory role in the immune system [2, 14, 44]. KLRG1 can inhibit the proliferative capacity and effector function of NK cells and T-cells by inhibiting AKT phosphorylation or enhancing AMPK phosphorylation [9, 42]. After viral infection, the ability of mouse NK cells to produce interferon-γ (IFN-γ) is negatively correlated with KLRG1 expression [14].

The functionality of KLRG1 is modulated in a complex manner by factors such as its expression level, activation state, inflammatory factors, and other costimulatory molecules. The inhibitory potential of KLRG1 directly correlates with its expression on cell surfaces [39]. KLRG1 expression is positively correlated with age [10] but negatively correlated with the ability of NK cells to produce the proinflammatory factor IFN-γ [7, 14]. Furthermore, interleukin-2 (IL-2) can induce the expression of KLRG1 on tissue-resident Treg cells, but the specific regulatory mechanisms involved are still unclear [71].

In addition, the expression level of KLRG1 significantly increased after virus infection in mice [3, 14]. Interestingly, the costimulatory molecules PD-1 and cytotoxic T lymphocyte-associated protein 4 (CTLA-4) may have potential regulatory mechanisms involving KLRG1 that influence the quantity and activity of target cells. For example, Taylor et al. reported that PD-1 signaling deficiency enhances signal transducer and activator of transcription (STAT)-5 activation, which increases the proliferation of KLRG1^+^ ILC-2 cells. Moreover, PD-1 antibody blockade also enhanced KLRG1^+^ ILC-2 cell quantity and antiparasitic helminth immune function [72]. CTLA-4, which binds to its ligand CD80, triggers the Hippo pathway, leading to Yes-associated protein degradation and consequently upregulating B lymphocyte-induced maturation protein 1 (Blimp-1) to promote terminal T-cell differentiation [73]. Blimp-1 is a transcription factor required for the differentiation of effector CD8^+^ T cells, and in the absence of Blimp-1, T cells fail to differentiate into KLRG1^hi^ IL-7R^low^ short-lived effector CD8^+^ T cells, resulting in reduced KLRG1 expression [74]. More research is needed to fully understand the relationship between KLRG1 and those costimulatory molecules. Moreover, the inhibitory effect of KLRG1 was negatively correlated with the expression level of the transferrin receptor on cell surfaces and decreased with increasing lymphocyte proliferation [75].

### The role of KLRG1 in diseases

#### KLRG1 in autoimmunity

Autoimmune diseases are a group of diseases in which the body develops an abnormal immune response to self-antigens, resulting in self-tissue damage [76]. As an immune checkpoint receptor, KLRG1 may play a role in autoimmune diseases by regulating the effector functions and proliferative capacities of T- and NK cells and controlling immune tolerance [77, 78]. Numerous studies have shown that KLRG1 expression is increased on NK and T-cell subsets in patients with a variety of autoimmune diseases, including primary biliary cholangitis (PBC), IBM, systemic lupus erythematosus (SLE), and experimental autoimmune encephalomyelitis (EAE) [18, 56, 79–81] but is reduced in NK cells in the peripheral blood of patients with SLE [15] and is lacking in the peripheral blood of patients with autoimmune lymphoproliferative syndrome (ALPS) [56]. In summary, increased KLRG1 expression mostly positively correlates with disease severity [15, 18, 80, 81] or increases with the degree of T-cell differentiation and is positively correlated with cytotoxicity (Table 2) [79, 82, 83]. For example, KLRG1^+^ T-cell infiltration in liver samples from PBC patients is positively correlated with severe histologic hepatic inflammation and histologic hepatic fibrosis, while in peripheral blood (PB) samples, KLRG1^+^ T-cells contain substantially greater levels of cytotoxic molecules (such as granzyme B and perforin), inflammatory cytokines (IFN-γ and tumor necrosis factor α (TNF-α)), and inflammatory chemokine receptors than their KLRG1-negative counterparts [18]. Increased expression of KLRG1 on Treg cells derived from the central nervous system was positively correlated with disease severity in a mouse model of EAE. KLRG1^+^ Treg cells have a more rapid cell cycle than KLRG1^−^ Tregs and produce more interleukin-10 with the ability to inhibit EAE, thereby modulating disease severity [81]. In SLE patients, the expression level of KLRG1 is significantly elevated in T-cells and is positively correlated with SLE disease activity [80]. Interestingly, in the PB of SLE patients, the expression level of KLRG1 is decreased in NK cells, which negatively correlates with SLE disease activity, but KLRG1 expression increases after in vitro hydroxychloroquine (HCQ) treatment [15]. Although the use of HCQ may be related to the expression level of KLRG1 in NK cells, the mechanism by which this drug works needs further research.

KLRG1 also plays a role in the progression of autoimmune diseases. In the nonobese diabetic mouse model of type 1 diabetes (T1D), KLRG1 is expressed on Foxp3^+^ Treg cells in the pancreatic islets and plays a role in inhibiting pancreatic autoimmunity, resulting in a decrease in the proliferative and inhibitory functions of Foxp3^+^ Treg cells. This absence of Foxp3^+^ Treg cells in pancreatic islets may promote T1D progression, and IL-2 treatment fails to reverse this deficiency [84]. Since KLRG1 inhibits mTOR signaling through the PI3K/AKT pathway, the lack of KLRG1 expression on TCRαβ^+^ CD4^−^ CD8^−^ double-negative T-cells in ALPS patients leads to overactivity of the mTOR pathway, resulting in abnormal lymphocyte proliferation [56]. In conclusion, these studies suggest that KLRG1 is mostly positively correlated with disease severity in autoimmune diseases [18, 81], can serve as a marker of disease progression in patients with IBM [27, 85] and SLE [80], and has potential as a therapeutic target in patients with IBM [79]. Targeting KLRG1^+^ lymphocytes may be a promising strategy for developing therapeutic agents for treating autoimmune diseases [18, 79].

**Table 2:** Expression and role of KLRG1 in autoimmune diseases

Disease	Species	Status	Sample	Cell	Control	KLRG1 expression (compared to control)	Results	References
PBC	Human	Newly diagnosed patient	Liver	CD8^+^ T	Hepatic hemangioma resection and orthotropic liver transplantation patients	High	KLRG1 expression level positively correlates with liver transplant risk and drives PBC autoimmune attack.	[18]
		Newly diagnosed patient	PB	CD8^+^ T	HC	High	KLRG1-positive T cells have increased cytotoxic molecules such as granzyme B and perforin, inflammatory cytokines (IFN-γ and TFN-α), and inflammatory chemokine receptors.	
IBM	Human	Newly diagnosed patient	Muscle tissues and PB	CD8^+^ T	Patients without neuromuscular disease (Muscle); HC (PB)	High	KLRG1 expression level increases with T-cell differentiation and positively correlates with cytotoxicity, allowing assessment of IBM progression and becoming a possible therapeutic target, and KLRG1-positive cells can invade IBM myofibers.	[79, 83]
T1D	Mouse	BDC2.5 (ICOS^−/−^)	Pancreatic islets	Treg	BDC2.5 (WT)	High	KLRG1^+^foxp3^+^ Treg cells are prone to apoptosis, have poor proliferation ability, reduce the function of inhibiting pancreatic autoimmunity, and promote the progression of T1D.	[84]
SLE	Human	Newly diagnosed patient	PB	CD8^+^ T	HC	High	KLRG1 expression level positively correlates with SLE disease activity, and KLRG1-positive CD8^+^ T cells have increased secretion of inflammatory cytokines, which promotes pro-inflammatory disease.	[80]
		Newly diagnosed patient	PB	NK	HC	Low	KLRG1 expression level negatively correlated with SLE disease activity, increased after treatment with hydroxychloroquine in vitro, and KLRG1-positive NK cells had decreased IFN-γ production.	[15]
EAE	Mouse	C57BL/6J (EAE)	Brain tissue	Treg	C57BL6/J (WT)	High	KLRG1 expression level positively correlated with disease severity, and KLRG1-positive cells have increased interleukin-10 and IFN-γ and decreased interleukin-17.	[81]
ALPS	Human	Newly diagnosed patient	PB	TCRαβ^+^ CD4^−^ CD8^−^ T	HC	Lack	KLRG1 deficiency leads to hyperactive mTOR pathway and abnormal lymphocyte proliferation.	[56]

*Abbreviations* HC, healthy control; PB, peripheral blood; WT, wild type

#### KLRG1 in infection

The amount of available data regarding the expression level and role of KLRG1 in infectious diseases, including viral, bacterial, and parasitic infections, is increasing (Table 3) [33, 50, 86, 87]. During infection, the TCR recognizes foreign antigen peptides present on the major histocompatibility complex, followed by the activation and proliferation of T-cells and the regulation of a variety of effector molecules to modulate the antipathogen immune response [88]. As an inhibitory receptor, KLRG1 regulates the activation and function of T-cells and NK cells through various pathways [50, 89, 90]. Sustained antigen stimulation during chronic viral infection leads to T-cell exhaustion characterized by progressive loss of effector function and increased expression of inhibitory immune checkpoint receptors [88, 91]. Research has shown that after infection with viruses, including HCV, human immunodeficiency virus (HIV), mouse cytomegalovirus (MCMV), chronic hepatitis B (CHB), and lymphocytic choriomeningitis virus (LCMV), the expression levels of KLRG1 on virus-specific CD8^+^ T-cells [92, 93] and NK cells [14, 19, 94] are elevated, and KLRG1 function increases after receiving repeated and sustained antigenic stimulation. However, regarding influenza viruses, only 40–73% of CD8^+^ cells specific for influenza epitopes are expressing KLRG1 [41].

KLRG1 can inhibit the proliferation and function of immune cells, providing a new target for viral immunotherapy. In HCV infection, KLRG1 inhibits immune cell proliferation and function through the AKT, p16^ink4a^ and p27^kip1^ pathways, and blocking these pathways may improve vaccine responses [11, 50]. Importantly, one study regarding HCV patients revealed that KLRG1 is a marker for the activation of memory NK cells, which can proliferate more efficiently when restimulated with HCV antigens, thereby facilitating the memory immune response; this finding highlights the potential of KLRG1^+^ memory NK cells to offer important insights for future vaccine design [94]. Hendrik Streeck et al. reported that the plasma level of soluble E-cad (sE-cad), which can interact with KLRG1^+^ HIV-1-specific CD8^+^ T-cells to inhibit IFN-γ secretion and antiviral activity, increased after HIV-1 infection [93]. Ex vivo antibody blockers targeting KLRG1 restored HIV-specific immune responses and the ability of NK cells to kill HIV-infected cells [19, 95]. During the early stage of MCMV infection, KLRG1^+^ NK cells in the spleen and liver proliferate; the expression of B-cell lymphoma-2 is selectively lost in KLRG1^+^ NK cells at the late stage of infection, leading to the apoptosis of KLRG1^+^ NK cells [14, 89, 96]. In addition, KLRG1^−^Ly49H^+^ NK cells preferentially expand and generate memory NK cells compared to KLRG1^+^Ly49H^+^ NK cells, indicating that during MCMV infection, Ly49H^+^ NK cells lose their potential to produce memory when they reach a mature stage of differentiation [33]. In CHB infection, KLRG1^+^ NK cells, the number of which is increased in PB and liver samples, can inhibit liver fibrosis by enhancing the apoptosis of activated hepatic stellate cells through the upregulation of expression of tumor necrosis factor-related apoptosis-inducing ligands. This antifibrotic function of KLRG1^+^ NK cells provides a new therapeutic approach for treating liver fibrosis in patients with CHB [97].

KLRG1 has also been detected in a few bacterial infections, such as *Mycobacterium tuberculosis* (Mtb) and *Helicobacter pylori* (*H. pylori*) [90, 98]. During Mtb infection, KLRG1 is overexpressed on lung CD4^+^ T-cells, and these KLRG1^+^CD4^+^ T-cells secrete significantly greater amounts of IFN-γ, IL-2, and tumor necrosis factor-alpha than do KLRG1^−^CD4^+^ T-cells [90]. Increased KLRG1^+^CD4^+^ T-cells may negatively impact immunity by enhancing stromal adherence and restricting the access of terminal effector cells to the infection site [90]. Blockade of KLRG1 enhances AKT signaling, reduces lung burden, and prolongs survival time after infection; thus, KLRG1 is a potential target for antituberculosis immunotherapy [99]. However, Park et al. conducted a transcriptomic analysis of *H. pylori*-infected cells before and after the use of kimchi extract and reported that *KLRG1* gene expression significantly decreased during *H. pylori* infection but increased after nutritional supplementation with kimchi extracts [98]. This is the first study to identify KLRG1 in *H. pylori* infection, and its role still needs further investigation.

Parasitic infection is a type of disease in which parasites invade and cause infection in humans or animals, leading to persistent infection mainly by inhibition of the immune response and generation of immune tolerance [100]. KLRG1 expression is upregulated during infection with several parasitic protozoans, including *Toxoplasma gondii*, *Nippostrongylus brasiliensis*, and *Leishmania* [32, 101]. This increase may inhibit the immune function of T-cells and limit the clearance of *T. gondii* [87, 102]. KLRG1 may play a role in immune regulation and tolerance by inhibiting immune cell proliferation and cytokine production [100]. KLRG1 can bind to ligands on the surface of ILC-2s, inhibit ILC-2 proliferation and activation, and reduce the ability of ILC-2s to produce cytokines, thereby decreasing the immune responses of ILC-2s to parasitic infection [49]. Antibody blockade of PD-1 during *N. brasiliensis* infection increases the number of KLRG1^+^ ILC-2s, which enhances the protective function of ILC-2s in parasitic infections and reduces the disease burden [72]. In aged visceral *Leishmania-*infected mice, the expression of KLRG1 is increased on hepatic and splenic CD4^+^ and CD8^+^ T-cells, leading to decreased IFN-γ production and reduced proliferative ability of T-cells, which suggests that senescence may increase the susceptibility of patients to visceral *Leishmania* infection [101]. In summary, KLRG1 expression is increased on immune cells after repeated and sustained antigenic stimulation and can serve as a marker for assessing the extent of infection in patients infected with HCV and Mtb [92, 99]. KLRG1 expression can also lead to persistent infections by inhibiting the proliferation and function of immune cells and can serve as a potential therapeutic target in patients with HCV, HIV, and Mtb [92, 95, 99]. Inhibition of KLRG1 expression may become a new method for treating infectious diseases [11, 86].

**Table 3:** Expression and role of KLRG1 in infectious diseases

Disease		Species	Status	Sample	Cell	Control	KLRG1 expression (compared to control)	Results	References
Viral infection	HCV	Human	Newly diagnosed patient	PB	Virus specific CD8^+^ T	Influenza patients	High	KLRG1 expression level increased with T cell differentiation and can serve as a marker of T cell differentiation.	[92]
			Newly diagnosed patient	PB	CD4^+^ T	HCV-uninfected patients	High	KLRG1-positive cells have decreased proliferative capacity and IL-2 secretion, and no response to HBV vaccine.	[11]
			Newly diagnosed patient	PB	NK	HC	High	KLRG1-positive NK cells are prone to apoptosis, have decreased proliferative capacity, IFN-γ secretion and cellular function, and can differentiate into memory cells.	[50, 94]
	HIV	Human	Newly diagnosed patient	PB	Antigen specific CD8^+^ T	HIV-uninfected patients	High	KLRG1-positive cells have decreased IFN-γ secretion and antiviral activity.	[93, 95]
			Newly diagnosed patient	PB, lymph nodes	NK	HC	High	KLRG1-positive cells have decreased IFN-γ secretion and cellular function.	[19]
	MCMV	Mouse	C57BL/6 (MCMV)	Spleen and liver	NK	C57BL/6 (WT)	High	KLRG1-positive cells have decreased proliferative capacity, IFN-γ, TNF-α, and NK cell-mediated cytotoxicity.	[14, 33, 89, 96]
	CHB	Human	Newly diagnosed patient	PB	NK	HC	High	KLRG1 expression level negatively correlated with serum alanine aminotransferase and azelaic transaminase levels, and KLRG1-positive cells have decreased interferon-gamma.	[97]
			Newly diagnosed patient	Liver	NK	Patients undergoing surgery for colorectal metastases or hepatocellular carcinoma	High	KLRG1-positive cells have increased IFN-γ, activate and induce hepatic stellate cell apoptosis, and inhibit hepatic stellate cell proliferation and CHB fibrosis.	[97]
	LCMV	Mouse	P14 chimeric (LCMV early)	Spleen	CD8^+^ T	P14 chimeric (LCMV systole)	High	KLRG1 expression level increased during early infection and decreased during systole, and KLRG1-positive cells have decreased Interleukin-7 receptor and proliferative capacity.	[68]
Bacterial infection	Mtb	Human	Newly diagnosed patient	Lung	CD4^+^ T	HC	High	KLRG1-positive cells have increased IFN-γ, IL-2, and TNF-α and are prone to apoptosis.	[90]
		Mouse	C57BL/6 (KLRG1^**−/−**^**)**	Lung	CD4^+^ T	C57BL/6 (WT)	Lack	KLRG1 lack results in decreased Mtb colony-forming units in the lungs, prolonged survival after infection, and increased levels of IFN-γ and TNF.	[99]
Parasitic infection	*Toxoplasma gondii*	Mouse	C57BL/6 (*Toxoplasma gondii*)	Spleen and liver	CD8^+^ T and CD4^+^ T	C57BL/6 (WT)	High	KLRG1-positive cells have decreased immune function.	[32, 102]
	*Nipostrongylus brasiliensis*	Mouse	C57BL/6 (Pdcd1^**−/−)**^	Lung	ILC-2s	C57BL/6 (WT)	High	KLRG1-positive cells have decreased proliferative capacity, activation levels, ability to produce cytokines, and immune responses to parasitic infections.	[72]
	*Leishmania*	Mouse	C57BL/6 (aged)	Spleen and liver	CD4^+^ T and CD8^+^ T	C57BL/6 (young)	High	KLRG1-positive cells have decreased proliferative capacity and IFN-γ.	[101]

#### KLRG1 in tumors

As protective factors of the human immune system, immune checkpoint molecules are critical for maintaining self-tolerance and modulating the duration and amplitude of physiological immune responses in peripheral tissues [103]. In tumor cells, the dysregulation of immune checkpoint proteins is an important mechanism of tumor immune resistance. When immune checkpoint molecules are overexpressed or overactivated, immune function is inhibited [104]. Research has shown that KLRG1 plays an important role in many types of tumors, including solid and malignant hematological tumors. KLRG1 can bind to ligands and play an important role in tumor development by regulating lymphocyte activity, inhibiting cytokine secretion, and inducing apoptosis to suppress the immune response [14, 50, 105]. In the tumor microenvironment, KLRG1 can inhibit the antitumor immune response and promote tumor escape [23, 105]. The expression level of KLRG1 is also significantly correlated with the immunotherapy responses of patients with various diseases and can serve as a biomarker for the prognoses of patients with tumors (Table 4) [16, 106, 107].

#### KLRG1 in solid tumors

In patients with solid tumors, KLRG1 may affect the proliferation of tumor cells or participate in the regulation of tumor immune escape and immune tolerance by interacting with cell surface receptor signaling pathways [16, 23, 108]. Compared to that in healthy populations, the expression of KLRG1 is increased in T-cells in patients with solid tumors such as those of breast and colorectal cancer (CRC) [8, 26, 107, 109] and decreased in NK cells in melanoma tumor tissues in mice and in lung tumor cells in patients with lung adenocarcinoma (LUAD) [16, 22, 28, 110]. The expression level of KLRG1 is positively correlated with antigen-presenting cell infiltration in LUAD, and Dietmar et al. reported that KLRG1^+^ effector CD8^+^ T-cells can differentiate into memory T-cells to promote antitumor immunity, which suggests that KLRG1 could be used in the development of mRNA vaccines [22, 111]. However, this antitumor effect of KLRG1 expression on tumor cells contradicts its protumor effect on immune cells [23]. Yang et al. reported that the expression of KLRG1 was significantly lower in lung tumor cells from LUAD patients than in those from healthy controls and that a decrease in KLRG1 expression enhanced the proliferation of LUAD cells. In addition, the expression of KLRG1 is positively correlated with the efficacy of immune checkpoint inhibitors, and patients with high KLRG1 expression have a better prognosis, which suggests that KLRG1 may become a prognostic biomarker for LUAD patients [16]. Yang et al. hypothesized that this contradiction may result from the competitive binding of KLRG1 on tumor cells to the ligand E-cad, which decreases the inhibitory effect of KLRG1 on T-cells and NK cells [16]. Therefore, in the study of the role of KLRG1 on tumor cells, KLRG1 expression levels on the surfaces of both immune cells and tumor cells should be measured.

KLRG1 can inhibit the antitumor activity of immune cells and promote tumor metastasis. In breast cancer patients, Yamauchi et al. reported that the interaction of E-cad and KLRG1 inhibits antibody-dependent cell-mediated cytotoxicity (ADCC), rendering human epidermal growth factor receptor-2-expressing tumor cells resistant to trastuzumab treatment. Removal of KLRG1-positive peripheral blood mononuclear cells can enhance trastuzumab-mediated ADCC activity and improve therapeutic efficacy, but the means by which this method enhances ADCC activity still needs further study [108]. NK cells have effective antitumor and antimetastatic activities [112]. However, breast cancer cells can reprogram tumor-exposed NK (teNK) cells to promote metastatic colony formation. Targeting KLRG1 expressed on teNK cells eliminates the metastasis-promoting effects of teNK cells and decreases colony formation, providing a new approach for preventing or treating metastatic tumors [8, 26]. Necroptosis is a form of necrotic programmed cell death that frequently occurs in advanced solid tumors and can inhibit the antitumor activities of T-cells and promote breast cancer metastasis by synergistically inhibiting the KLRG1 receptor [113]. In a mouse model of breast cancer, antibody neutralization of KLRG1 significantly increased the antitumor activities of tumor-infiltrating T-cells and PB T-cells and significantly reduced lung metastasis [24]. In mouse melanoma-related NK cells, a decrease in KLRG1 expression leads to a decreased proliferative capacity of intratumoral NK cells [110].

In addition, the expression level of KLRG1 is positively correlated with therapeutic outcome, which suggests that KLRG1 could serve as a marker to monitor the antitumor immune response induced by this therapy [28]. Antibodies that block CTLA-4 expression or activate 4-1BB both enhance the body’s antitumor immunity but fail to cure poorly immunogenic B16 melanomas when used alone [114, 115]. Curran et al. reported that the combined use of these two antibodies led to high expression of KLRG1 on tumor-infiltrating effector T-cells in mice, which promoted an immune-rejection response to melanoma [28]. In CRC patients, the mRNA expression level of *KLRG1* was significantly greater in tumor tissues than in paired normal tissues and tended to increase in the advanced stages of the disease [107]. Furthermore, KLRG1^+^ cytotoxic T-cells are enriched in CRC patients with a good prognosis [109], and CD27^low^KLRG1^+^ NK cells protect T-box expressed in T-cells (T-bet)-deficient mice from pulmonary metastatic colorectal carcinoma [116]. Overall, KLRG1 can inhibit the antitumor activities of immune cells, promote tumor metastasis, and lead to immune dysfunction in patients. KLRG1 in LUAD and melanoma can serve as a marker for detecting the response to treatment with immune checkpoint inhibitors [16, 28] and has the potential to be a therapeutic target in breast cancer, melanoma, and CRC [8, 22, 116].

#### KLRG1 in hematological malignancies

HMs are a group of hematopoietic diseases characterized by a high degree of malignancy, complex treatment, and poor prognosis. KLRG1 expression is increased on a variety of immune cells in patients with a variety of HMs, including chronic lymphocytic leukemia (CLL), follicular lymphoma (FL), acute myeloid leukemia (AML), and multiple myeloma (MM) [117–119]. KLRG1-positive cells have impaired proliferation ability and can bind to ligands to inhibit CD8^+^ T-cell effector function, leading to immune dysfunction in patients [117–121]. In CLL patients, the expression levels of KLRG1^+^CD8^+^ T-cells and plasma sE-cad are increased. Based on data obtained by Streeck et al. studying HIV, these two proteins may interact to inhibit KLRG1^+^CD8^+^ T-cell effector function, leading to immune dysfunction in CLL patients [117]. In patients with FL, CD8^+^ T-cells lose their proliferative capacity after differentiating into KLRG1^+^CD127^−^CD8^+^ T-cells, which have a greater capacity to produce cytokines but lower activity than KLRG1^−^CD127^+^CD8^+^ T-cells. Therefore, the modulation of CD8^+^ T-cell differentiation in FL by PI3K inhibitors may promote a more effective antitumor immune response and thus improve the clinical prognosis of lymphoma patients [121].

KLRG1 may also be a marker for monitoring antitumor responses induced by anti-4-1BB mAbs. In an Em-myc lymphoma model, anti-4-1BB mAb treatment induces KLRG1 expression in CD8^+^ T-cells [122]. A study of a combination treatment for AML including a vaccine and an anti-4-1BB mAb revealed that treatment-induced KLRG1^+^ effector CD8^+^ T-cells were most effective for controlling disease progression [123]. In addition, in tumor-bearing mice, the migration of KLRG1^+^ NK cells to the bone marrow is impaired and regulated by C-X-C motif chemokine receptor 3, resulting in a rapid and selective decrease in the number of KLRG1^−^ NK cells with potent effector functions in the bone marrow, which contributes to tumor escape from NK cell-mediated immune surveillance [124]. In conclusion, KLRG1 has a similar role in hematologic malignancies as in solid tumors.

**Table 4:** Expression and role of KLRG1 in tumors

	Disease	Species	Status	Sample	Cell	Control	KLRG1 expression (compared to control)	Results	References
Solid tumor	Breast cancer	Human	Newly diagnosed patient	PB	CD4^+^ T and CD8^+^ T	HC	High	KLRG1-positive cells have increased expression of effector cytokines, granzyme, and perforin.	[8, 125]
	CRC	Human	Newly diagnosed patient	Tumor tissue	T	HC	High	KLRG1 expression level increased in patients with a high degree of tumor budding, which may lead to poor disease prognosis and metastasis in CRC patients.	[107]
		Mouse	BALB/C (T-bet^**−/−**^)	Spleen	NK	BALB/C (WT)	Low	KLRG1^+^ NK cells protect T-bet-deficient mice from pulmonary metastatic colorectal carcinoma.	[116]
	Melanoma	Mouse	C57BL/6 (Melanoma)	Tumor tissue	NK	C57BL/6 (Melanoma), spleen	Low	The expression level of KLRG1 positively correlated with the treatment effect, and KLRG1-positive cells have decreased proliferative capacity.	[110]
	LUAD	Human	Newly diagnosed patient	Lung	tumor	HC	Low	KLRG1-positive lung tumor cells have increased proliferative capacity.	[16]
HM	CLL	Human	Newly diagnosed patient	PB	CD8^+^ T	HC	High	KLRG1 binds to sE-cadherin impairs CD8^+^ T cell cytokine secretion and antiviral responses.	[117]
	FL	Human	Newly diagnosed patient	PB	CD8^+^ T	HC	High	KLRG1-positive cells have decreased cell proliferation capacity and activity, and increased IFN-γ, TNF-α, granzyme B, and perforin.	[121]
	AML	Human	Newly diagnosed patient	PB and bone marrow	NK	HC	High	KLRG1 can block the anti-leukemia function of NK cells.	[118]
	MM	Human	Newly diagnosed patient	PB and bone marrow	Cytotoxic T	HC	High	The function of KLRG1-positive cytotoxic T cells is impaired.	[120]
		Mouse	C57BL/KaLwRij (MM)	Bone marrow	NK	C57BL/KaLwRij (WT)	Low	Decreased KLRG1^−^ NK cells, KLRG1-negative NK cells have decreased and impaired transit to the bone marrow that helps tumors evade NK cell-mediated immune surveillance.	[124]

#### KLRG1 as a tool for immunotherapy

The direct recognition, rapid activation, and cytotoxicity of KLRG1 on cancer cells make it an attractive tool for cancer immunotherapy, and this has recently been extensively reviewed [5, 70]. KLRG1 has demonstrated significant antitumor and inhibitory effects on tumor growth in a wide range of malignant tumors, while KLRG1-targeted inhibitors have also been developed as tumor immunotherapies and have been a popular area of research in immunotherapy [8, 23–25].

The absence of KLRG1 signaling alone significantly reduced the growth of melanoma and breast cancer tumors in mouse lungs. In a 4T1 breast cancer model, an anti-KLRG1 antibody inhibited the binding of mouse E-cad to KLRG1 and significantly reduced lung metastasis [8]. In a mouse model of breast cancer, antibody neutralization of KLRG1 reduced the formation of tumor colonies [26], significantly increased the antitumor activity of tumor-infiltrating cells and peripheral T-cells, and reduced lung metastasis [24].

In addition, combinations of checkpoint blockade therapies have shown effectiveness in many different types of cancer [126]. Tregs can hinder T-cell function in various tumors and inhibit antitumor immunity [127, 128]. In a melanoma mouse model, the use of an anti-KLRG1 antibody alone moderately depleted intratumoral Tregs but not peripheral Tregs, which prevented the autoimmune side effects caused by systemic depletion of Tregs [25, 129]. Administration of a bromodomain inhibitor also partially depleted intratumoral Tregs, and when this treatment is combined with anti-KLRG1 antibody, tumor-infiltrating CD8^+^ T-cells express higher levels of granzyme B and IFN-γ, significantly improving the antitumor response [25]. KLRG1 blockade works synergistically with PD-1 checkpoint therapy, which increases the frequency and maturation of CD8^+^ T-cells and NK cells in the tumor microenvironment, promoting antitumor immunity against melanoma tumor growth [23]. In a mouse model of breast cancer, the combination of an anti-KLRG1 antibody with a DNA methyltransferase inhibitor further reduced the metastatic potential of breast cancer and effectively prevented metastatic recurrence compared to use of the antibody alone [26]. Tumors that do not respond to anti-PD-1 monoclonal antibody therapy alone may still benefit from combination therapy with KLRG1 blockade [23]. In MC38 colon cancer and B16F10 melanoma models, combination therapy of anti-KLRG1 and anti-PD-1 antibodies inhibited tumor growth and synergistically reduced tumor volume more than treatment with anti-KLRG1 or anti-PD-1 antibody controls alone [8].

## Conclusions

Studies targeting KLRG1 have shown that KLRG1 not only serves as a marker of T-cell senescence [65] but also increases with disease severity in autoimmune, viral infections and cancer, and can serve as a biomarker for assessing disease progression and prognosis [16, 27, 80, 92, 99]. Recently, researchers have revealed that targeting cells expressing KLRG1 has the potential to control disease progression by attenuating the inhibitory effects of antitumor responses, thereby benefiting the host. As discussed above, KLRG1-related signal transduction occurs mainly through the PI3K/AKT, KLRG1/AMPK, and p16^ink4a^/p27^kip1^ pathways to inhibit the cytotoxicity and proliferation of NK cells and T-cells, cytokine secretion, and telomerase expression and activity [10–12]. More work is needed to investigate the roles of additional regulatory mechanisms or regulatory mechanisms between different inhibitory receptors in diverse cellular contexts to further explain why tumors that are insensitive to other inhibitor therapies could still benefit from combination therapy with an anti-KLRG1 antibody. Indeed, the combination of anti-KLRG1 with other inhibitors can improve the antitumor response in mice with melanoma, further reducing the metastatic potential of breast cancer and effectively preventing metastatic recurrence [23, 26]. Although there are no approved anti-KLRG1 drugs on the market, Ulviprubart (ABC008), an anti-KLRG1 drug product developed by Abcuro for the treatment of IBM, has progressed to clinical phase 2/3 and has been granted orphan drug status by the U.S. Food and Drug Administration and the European Medicines Agency [27]. The study of ABC015 for the treatment of cancer is in the preclinical stage. It is foreseeable that the emergence of future anti-KLRG1 drugs will lead to the development of new treatment strategies for tumor suppressor receptor immunotherapy.

## Data Availability

No datasets were generated or analysed during the current study.

## Abbreviations

KLRG1: Killer cell lectin-like receptor G1
ITIM: Immunoreceptor tyrosine-based inhibitory motif
NK: Natural killer
Treg: Regulatory T
APCs: Antigen-presenting cells
TCR: T cell receptor
AMPK: AMP-responsive protein kinase
HM: Hematological malignancies
mAb: Monoclonal antibody
IBM: Inclusion body myositis
mKLRG1: Mouse KLRG1
hKLRG1: Human KLRG1
rKLRG1: Rat KLRG1
PD-1: Programmed cell death protein 1
PI3K: Phosphoinositide 3-kinase
E-cad: E-cadherin
N-cad: N-cadherin
R-cad: R-cadherin
ILC-2s: Type 2 innate lymphoid cells
PIP_2_: Phosphatidylinositol (4,5) bisphosphate
PIP_3_: Phosphatidylinositol (3,4,5) bisphosphate
mTOR: Mammalian target of rapamycin
HCV: Hepatitis C virus
FOXO: Forkhead box O
CDK: Cyclin-dependent kinase
PP2C: Protein Phosphatase 2 C
SHIP-1: Src Homology 2-containing Inositol Phosphatase-1
SHP-2: Src homology-2-containing protein tyrosine phosphatase 2
IFN-γ: Interferon-γ
IL-2: Interleukin-2
CTLA-4: Cytotoxic T lymphocyte-associated protein 4
Blimp-1: B lymphocyte-induced maturation protein 1
PBC: Primary biliary cholangitis
T1D: Type 1 diabetes
SLE: Systemic lupus erythematosus
EAE: Experimental autoimmune encephalomyelitis
ALPS: Autoimmune lymphoproliferative syndrome
PB: Peripheral blood
HC: Healthy control
TNF-α: Tumor necrosis factorα
HCQ: Hydroxychloroquine
HIV: Human immunodeficiency virus
MCMV: Mouse cytomegalovirus
CHB: Chronic hepatitis B
LCMV: Lymphocytic choriomeningitis virus
sE-cad: Soluble E-cadherin
Mtb: Mycobacterium tuberculosis
H. Pylori: Helicobacter pylori
CRC: Colorectal cancer
LUAD: Lung adenocarcinoma
ADCC: Antibody-dependent cell-mediated cytotoxicity
TeNK: Tumor-exposed NK
T-bet: T-box expressed in T cells
CLL: Chronic lymphocytic leukemia
FL: Follicular lymphoma
AML: Acute myeloid leukemia
MM: Multiple myeloma
